# A phase I study to assess safety, pharmacokinetics, and pharmacodynamics of a vaginal insert containing tenofovir alafenamide and elvitegravir

**DOI:** 10.3389/fcimb.2023.1130101

**Published:** 2023-04-19

**Authors:** Andrea R. Thurman, Louise A. Ouattara, Nazita Yousefieh, Peter L. Anderson, Lane R. Bushman, Xi Fang, Homaira Hanif, Meredith Clark, Onkar Singh, Gustavo F. Doncel

**Affiliations:** ^1^ CONRAD, Eastern Virginia Medical School, Norfolk and Arlington, VA, United States; ^2^ University of Colorado, Colorado Antiviral Pharmacology Lab, School of Pharmacy, Anschutz Medical Campus, Aurora, CO, United States

**Keywords:** vaginal insert, tenofovir alafenamide, elvitegravir, microbicide agent, HIV prevention

## Abstract

**Background:**

New multi-purpose prevention technology (MPT) products are needed to prevent human immunodeficiency virus (HIV) and herpes simplex virus type 2 (HSV2). In this study, we evaluated a fast-dissolve insert that may be used vaginally or rectally for prevention of infection.

**Objective:**

To describe the safety, acceptability, multi-compartment pharmacokinetics (PK), and *in vitro* modeled pharmacodynamics (PD) after a single vaginal dose of an insert containing tenofovir alafenamide (TAF) and elvitegravir (EVG) in healthy women.

**Methods:**

This was a Phase I, open-label, study. Women (n=16) applied one TAF (20mg)/EVG (16mg) vaginal insert and were randomized (1:1) to sample collection time groups for up to 7 days post dosing. Safety was assessed by treatment-emergent adverse events (TEAEs). EVG, TAF and tenofovir (TFV) concentrations were measured in plasma, vaginal fluid and tissue, and TFV-diphosphate (TFV-DP) concentration in vaginal tissue. PD was modeled *in vitro* by quantifying the change in inhibitory activity of vaginal fluid and vaginal tissue against HIV and HSV2 from baseline to after treatment. Acceptability data was collected by a quantitative survey at baseline and post treatment.

**Results:**

The TAF/EVG insert was safe, with all TEAEs graded as mild, and acceptable to participants. Systemic plasma exposure was low, consistent with topical delivery, while high mucosal levels were detected, with median TFV vaginal fluid concentrations exceeding 200,000 ng/mL and 1,000 ng/mL for up to 24 hours and 7 days post dosing, respectively. All participants had vaginal tissue EVG concentrations of > 1 ng/mg at 4 and 24 hours post dosing. The majority had tissue TFV-DP concentrations exceeding 1000 fmol/mg by 24 – 72 hours post dosing. Vaginal fluid inhibition of HIV-1 and HSV-2 *in vitro* significantly increased from baseline and was similarly high at 4 and 24 hours post dosing. Consistent with high tissue TFV-DP concentrations, p24 HIV antigen production from ectocervical tissues infected *ex vivo* with HIV-1 significantly decreased from baseline at 4 hours post dosing. HSV-2 production from tissue also decreased post treatment.

**Conclusions:**

A single dose of TAF/EVG inserts met PK benchmarks, with PK data supporting an extended window of high mucosal protection. PD modeling supports mucosal protection against both HIV-1 and HSV-2. The inserts were safe and highly acceptable.

**Clinical trial registration:**

ClinicalTrials.gov, identifier NCT03762772.

## Introduction

Over 37 million people worldwide are infected with human immunodeficiency virus type 1 (HIV-1) (United Nations, 2018). Adolescent girls and young women (AGYWs) bear the burden of the HIV-1 pandemic, with more than 59% of new infections occurring in women in sub-Saharan Africa (United Nations, 2018). Genital herpes is a risk factor for HIV-1 acquisition and transmission, and it is highly prevalent globally and particularly in women (Looker et al., 2017; Maughan-Brown et al., 2019). An estimated 491 million people aged 15–49 (13%) worldwide have HSV-2 infection (WHO, 2022). Besides condoms, there is no biomedical prevention tool for genital herpes.

Development of safe, effective, and acceptable microbicides and pre-exposure prophylaxis (PrEP) products can successfully reduce HIV-1 sexual transmission in young women (Abdool Karim et al., 2010; Baeten et al., 2012; Baeten et al., 2016). Currently, oral tenofovir disoproxil fumarate (TDF) 300 mg in combination with emtricitabine (FTC) 200 mg (Truvada®) is approved for prevention of HIV-1 acquisition in men and women in the United States and several other countries worldwide (Grant et al., 2010; Baeten et al., 2012; Thigpen et al., 2012). The daily dosing requirement and systemic side effects, primarily gastrointestinal (GI), among other barriers including stigma and lack of support from family or partner, have made it difficult, particularly for AGYWs, to adhere to daily oral TDF/FTC for HIV-1 prevention (Van Damme et al., 2012; Magazi et al., 2014; van der Straten et al., 2014b; Corneli et al., 2015; Marrazzo et al., 2015; Corneli et al., 2016; Amico et al., 2017; Joseph Davey et al., 2022; Tapsoba et al., 2022). Oral tenofovir alafenamide (TAF) combined with FTC (Descovy®), which shows fewer side effects, was recently approved by the United States Food and Drug Administration for HIV-1 prevention, but only among individuals whose primary risk of HIV-1 acquisition is not through vaginal exposure (Mayer et al., 2020).

Topical tenofovir (TFV), as a 1% vaginal gel, was effective in reducing the incidence of HIV-1 (Abdool Karim et al., 2010) and herpes simplex virus type 2 (HSV-2) (Marrazzo et al., 2014) in the CAPRISA 004 prevention trial. However, adherence to both daily and peri-coital dosing regimens of TFV 1% gel was poor, particularly among AGYW, in subsequent Phase II and III trials (Marrazzo et al., 2015; Delany-Moretlwe et al., 2018). Potential reasons for decreased adherence included the stigma and inconvenience of storing and carrying single-dose, plastic vaginal gel applicators, the vaginal discharge associated with gel use, and the complicated two-dose, peri-coital, “BAT 24” dosing regimen (van der Straten et al., 2012; Succop et al., 2014; van der Straten et al., 2014a; Montgomery et al., 2018; Scorgie et al., 2018).

However, vaginal or rectal administration of pharmaceuticals and PrEP products provides local absorption of drug, resulting in enhanced bioavailability and high concentrations at the portal of virus entry, with limited systemic exposure, decreasing systemic side effects, which may increase adherence (Alexander et al., 2004; Hussain and Ahsan, 2005; Thurman et al., 2013; Baeten et al., 2016). Based on feedback from end users and with the goal of extending the window of protection and making its use more convenient, we selected a combination of two synergistic and mechanistically different antiretrovirals, a potent prodrug of the nucleotide TFV, tenofovir alafenamide (TAF), and an integrase inhibitor, elvitegravir (EVG) (Lampiris, 2012; Kulkarni et al., 2014; Elliot et al., 2016). EVG acts against HIV-1 by strand transfer inhibition preventing translocation of the reverse transcribed viral DNA into the nucleus and subsequent integration into the genome (Lampiris, 2012; Mohideen et al., 2017). *In vitro* data indicate the EC_50_ of EVG is less than 1 nM, which corresponds to about 0.5 – 1.0 ng/mL (Shimura et al., 2008; Ramanathan et al., 2011; Massud et al., 2015). In rhesus macaques, after one oral dose of EVG, raltegravir or dolutegravir, animals receiving EVG had the highest rectal and vaginal mucosal concentrations of drug (Massud et al., 2015), with peak concentrations of EVG in vaginal fluid of over 48,000 ng/mL by 24 hours post dose.

We previously treated cervico-vaginal (CV) explant tissues at varying times pre, during and post exposure to HIV-1_BaL_ *in vitro* and measured p24 antigen production for each drug at the various exposure times (Ouattara et al., 2016). We found that both EVG and TAF reduced p24 antigen production at all time points tested, up to 24 hours pre-exposure and up to 48 hours post exposure, providing an ideal combination to potentially extend the window of protection around a single use (Ouattara et al., 2016). From these *ex vivo* modeling experiments, we targeted an EVG tissue concentration of 1000 ng/g (1 ng/mg) as our benchmark for protection. Regarding TAF, which is converted intracellularly into TFV-DP, we adapted the benchmark concentration of 1000 fmols/10^6^ cells established in a SHIV vaginal challenge study in macaques (Dobard et al., 2022). Two separate SHIV vaginal and rectal challenge studies in nonhuman primates have demonstrated that the TAF/EVG inserts, applied vaginal or rectally, significantly prevent infection (Dobard et al., 2022; Makarova et al., 2022). Furthermore they prevented infection being administered before (pre-exposure) or after (post-exposure) repeated vaginal viral challenges (Dobard et al., 2022).

The co-primary objectives of the current study as stated in the protocol were to evaluate genital and systemic safety, and describe multi-compartmental pharmacokinetics (PK) after a single vaginal dose of the TAF/EVG vaginal insert in healthy, HIV-1 uninfected women. The secondary objectives of this study were to model pharmacodynamics (PD) *in vitro* against both HIV-1 and HSV-2 in both CV fluid and tissue. Additional secondary objectives were to assess insert disintegration *in vivo* and to explore user’s experiences with and acceptability of the vaginal product. Finally the exploratory objective of this study was to model anti-HSV2 activity ex vivoin CV tissue.

## Materials and methods

### Clinical study

CONRAD 146 was a Phase I, open-label, randomized, parallel-group study conducted at the CONRAD Intramural Clinical Research Center at Eastern Virginia Medical School (EVMS) (Norfolk, VA). The study was approved by the Advarra (central, independent) Institutional Review Board (Pro00030334) under a waiver of oversight from EVMS and registered with ClinicalTrials.gov (#NCT03762772). All participants signed written informed consent prior to any study procedures. We screened healthy women, 18 – 50 years old, who were HIV-1 uninfected, at low risk of sexually transmitted infections (STIs) and were protected from pregnancy during the study (by abstinence from sexual activity or consistent use of hormonal contraception (except depot medroxyprogesterone acetate (DMPA), due to its potential impact on study PK and PD endpoints (Zalenskaya et al., 2018; Thurman A. et al., 2019; Thurman AR. et al., 2019) or the contraceptive vaginal ring, as we did not want the presence of a vaginal ring to confound mucosal safety assessments), copper intrauterine device, sterilization of the participant or her sexual partner, or consistent condom use. Volunteers who were in a sexual relationship had to report that the relationship was mutually monogamous with a partner who was not known to be HIV positive and had no known risk of STI acquisition. We excluded women who were pregnant, had a current or recent (past 3 months) sexually transmitted infection (including *Trichomonas vaginalis*, *Neisseria gonorrhea*, *Chlamydia trachomatis*, HIV-1, and Hepatitis B) or symptomatic bacterial vaginosis. Women were excluded if they had chronic or acute vulvar or vaginal symptoms or a coexisting medical condition, which potentially made participation in the study less safe (e.g. a blood disorder that could lead to prolonged bleeding after genital biopsies). Because we assessed mucosal anti-viral activity against HIV-1 and HSV-2, participants could not be taking antivirals, anti-inflammatory drugs or systemic corticosteroids.

The study visits are summarized in **Supplemental Table 1**, but in brief, participants underwent 5 visits. Once eligibility was confirmed, participants completed a baseline questionnaire and provided baseline vaginal fluid and two ectocervical biopsies at Visit 2 (V2), which took place in the luteal phase of the menstrual cycle for participants not using contraceptive hormones. At Visit 3 (V3) participants inserted a single TAF/EVG vaginal insert. Depending on the participant’s group assignment, they had blood, vaginal fluid and vaginal tissue collected for PK at 4 or 24 hours post dosing. For PD endpoints, we collected vaginal fluid and two ectocervical biopsies for *ex vivo* HIV-1 or HSV-2 inhibition and infection assays at 4 or 24 hours for Group 1 and Group 2, respectively. At 4 or 24 hours post dosing, we inspected the vaginal vault to assess disintegration of the vaginal insert and the participant completed a post-dosing questionnaire. At Visit 4 (V4), 48 or 72 hours after dosing, for Groups 1 and 2 respectively, we collected blood, vaginal fluid and vaginal tissue for PK from all participants. Finally at Visit 5 (V5), which occurred 7 (± 2 days) after dosing, we obtained vaginal fluid from all participants for PK assessment.

### Adherence

We asked participants to refrain from vaginal or anal intercourse, douching, and use of all intravaginal objects and products starting 48 hours before baseline sampling at V2 through 5 days after the visit, and again starting 48 hours before V3 through 5 days after the last biopsy collection. We confirmed the absence of vaginal semen at V2 and V3 with a point-of-care prostate specific antigen test (ABA Card, Abacus Diagnostics, West Hill, CA). All participants inserted the vaginal product in the clinic.

### Randomization assignment

Participants were randomized in a 1:1 manner to have PD and PK tissue sampling obtained at 4 and 48 hours (Group 1) versus 24 and 72 hours (Group 2) post insert use. Participants in Group 1 had their 4 hour ectocervical tissue biopsies assessed for *in vitro* p24 antigen production after *ex vivo* HIV-1_BaL_ infection, while participants in Group 2 had their 24 hour post dosing ectocervical tissue assessed for HSV-2 DNA production after *ex vivo* HSV-2 infection. All participants had vaginal tissue collected for TAF, EVG, TFV and TFV-diphosphate (TFV-DP) concentrations. Designated research staff provided sample collection time assignments to participants per the plan above. There were no allocation errors.

### Study product

TAF/EVG vaginal inserts were manufactured under good manufacturing practices at Patheon Pharmaceuticals Inc., (Whitby, ON, Canada) as previously described (Peet et al., 2019). The active pharmaceutical ingredients were supplied by Gilead Sciences, Inc. (Foster City, CA, USA). The unit TAF dose was 20 mg and the unit EVG dose was 16 mg.

### Safety assessments

We assessed safety by treatment emergent adverse events (TEAEs). TEAEs were collected by the research coordinator at each study visit after genital sampling at baseline and graded for severity using the NIH DAIDS Grading Table for the Severity of Adult and Pediatric Adverse Events (Version 2.1, July 2017) and the Addendum 1, Female Genital Grading Table for Use in Microbicide Studies (Version 1.0, November 2007). Relationship of the TEAE to study product or study procedures was also determined by the study investigators.

### Assessment of plasma, vaginal fluid and tissue pharmacokinetic parameters

Briefly, a reversed-phase ultra-performance liquid chromatographic (UPLC), tandem mass spectrometry (MS/MS) assay for the determination of TFV and TAF was developed and validated for use with K_2_ EDTA anticoagulant plasma matrix and applied to vaginal fluid and vaginal tissue alternative matrices. The method utilizes stable labeled internal standards for each of the monitored compounds. The TFV concentration ranges were 0.05ng/mL to 10.0ng/mL and 0.500ng/mL to 500ng/mL. The TAF concentration ranges were 0.025ng/mL to 5.00ng/mL and 0.250 ng/mL to 250ng/mL.

Application to vaginal fluid alternative matrix collected *via* swab utilized K_2_ EDTA plasma as an extraction “solvent” and diluent to produce TFV and TAF concentrations within the analytical ranges validated for the plasma assay. Similarly, application to vaginal tissue alternative matrix collected *via* pinch biopsy utilized 70:30 MeOH:25 mM ammonium phosphate buffer (pH 7.4) as an extraction and homogenization solvent further diluted with K_2_ EDTA plasma to produce TFV and TAF concentrations within the analytical range of the validated plasma assay. Dilutions up to 10,000 fold were required with the resulting matrix for analysis generally >90% plasma and including dilution approaches 99% plasma. The K_2_ EDTA plasma diluted alternative matrix samples were subjected to the validated plasma assay.

A reversed-phase high performance liquid chromatographic (HPLC), tandem MS/MS assay for the determination of EVG in human plasma was developed and validated for use with K_2_EDTA anticoagulant plasma matrix and applied to vaginal fluid and tissue alternative matrices. The method utilizes a stable labeled internal standard. The assay utilizes a linear (1/x^2^ weighted) fit in the range of 0.025 ng/mL to 50.0 ng/mL. The assay has a minimum quantifiable limit of 0.025 ng/mL when 0.100 mL of human plasma is analyzed.

Similar to TAF and TFV, application to vaginal fluid alternative matrix collected *via* swab utilized K_2_ EDTA plasma as an extraction “solvent” and diluent to produce EVG concentrations within the analytical range validated for the plasma assay. Similarly, application to tissue alternative matrix collected *via* pinch biopsy utilized 70:30 lithium heparin plasma:collagenase solution as homogenization solvent further diluted with K_2_ EDTA plasma to produce EVG concentrations within the analytical range of the validated plasma assay. Dilutions up to 10,000 fold were required with the resulting matrix for analysis generally >90% plasma and including dilution approaches 99% plasma. The K_2_ EDTA plasma diluted alternative matrix samples were subjected to the validated plasma assay. Additional details may be found in **Supplemental Material**.

### Pharmacodynamic *in vitro* modeling of inhibition of HIV-1 infection in cells by vaginal fluid

TZM-bl cells (Wei et al., 2002) were plated and vaginal fluid (1:5 final dilution) with or without HIV was applied to the appropriate wells. For toxicity testing, 100 µL of medium with or without nonoxynol-9 or vaginal fluid was added to each well for 48h. The media was removed and replaced with 20µl of CellTiter 96® Aqueous One Solution Cell Proliferation Assay (Promega, Madison, WI, USA) and 100µl of cDMEM media for 3-4h. Absorbance was read at 490 nm. For efficacy (inhibition) testing, we used the Bright-Glo Luciferase Assay System (Promega, Madison, WI, USA) following the manufacturer’s instructions. Briefly, 100 µL of medium +/- vaginal fluid containing HIV-1_BaL_ (5x10^3^ TCID_50_) was added to each well. After 48h, the cells were lysed with 100 μL of Glo Lysis buffer. Lysate (50 µL) was transferred in a 96 wells black microtiter plate and 50 μL of Bright-Glo assay reagent was added and the luminescence was measured. The average percent inhibition of HIV-1_BaL_ growth in 3 wells with application of vaginal fluid, compared to control application of growth medium was reported for each participant. A comparison of vaginal fluid collected at baseline and after product insertion was also done for each participant.

### p24 antigen production after *ex vivo* infection with HIV-1_BaL_

Two ectocervical tissue biopsies were collected at baseline and placed immediately in two separate sterile cryovials filled with chilled RPMI 1640 media (Life Technologies, Carlsbad, CA) containing 10% fetal bovine serum (ATCC, Manassas, VA) and 100 U/ml penicillin and 100 µg/ml streptomycin (Thermo Fisher Scientific, Waltham, MA) (cRPMI) (Ouattara et al., 2022). Two ectocervical biopsies were obtained again 4 hours after the single vaginal insert dose. Biopsies were exposed to HIV-1_BaL_ (5x10^4^ TCID_50_/mL), within 30 minutes of collection, in presence of Interleukin-2 human (hIL-2) (Roche Diagnostics GmbH) at a final concentration of 100 U/mL. Ectocervical biopsies were washed 2 – 3 hours after viral exposure and then cultured in cRPMI media (500 µL) containing IL-2 for 21 days. We collected the tissue culture supernatant (approximately 300 µL) every three to four days and replenished cultures with media containing IL-2. We stored the supernatants at -80C and evaluated them at the end of the culture for HIV-1 p24 antigen expression by ELISA (Perkin Elmer, Waltham, MA, USA) in pg/mL. Area under the curve (AUC), cumulative (CUM) p24 antigen production, p24 production at Day 21 of tissue culture (p24_D21) were calculated as previously reported (Richardson-Harman et al., 2009).

### Viral replication after ex vivo infection with HSV-2

Two ectocervical biopsies were obtained at baseline and 24 hours after insert dosing. Ectocervical biopsies were placed into 1ml of explant media RPMI 1640 (Gibco, 11875093) 10%FBS, 100u/ml Pen/Strep (Gibco, 15140122) in the clinic. The biopsies were infected with 4X10^4^ PFU HSV-2-g strain (ATCC, VR-734) overnight in 48 well plate. The biopsies were washed 4 times with fresh media the following day. The media used for the fifth wash was saved as day 0 supernatant. Then 250 µl fresh media was added to the biopsies and incubated. On days 3, 6, 9 and 12, 125 µl of supernatant was removed and 140 µl of fresh media was added to the biopsy. All the supernatants were stored in -20 freezer until they were analyzed by polymerase chain reaction (PCR).

To perform the PCR the supernatants were thawed on ice. The PCR kit was purchased from Roche (catalogue number: 12239264001) and was used according to manufacturer’s protocol. HSV-2 DNA was evaluated by a quantitative PCR amplification of tissue culture supernatants using SYBR-green. Supernatants (6 μl) were amplified using the forward primer 5′- TCGCCAGCACAAACTCAT -3′ and the reverse primer 5′- CCACCGACCTCAAGTACAAC-3′ targeting glyprotein B. All data were normalized to day 0.

### Acceptability assessments

Prior to dosing, at baseline (visit 2) participants completed a 6 question, quantitative questionnaire, which assessed their baseline experiences with vaginal product use. At 4 or 24 hours post use, depending on their randomization assignment, participants completed a 13 question, quantitative survey about their experience using the single dose. Both surveys were completed at the clinic and participants were given a private space to read and complete the questionnaires. The acceptability survey was adapted from a previously used assessment from a study of a tenofovir/emtricitabine vaginal insert (Mauck et al., 2015), but was not otherwise validated.

### Ethics and trial registration

The study was conducted following the ethical standards outlined in the Helsinki Declaration (1983). The study was approved by the Advarra Institutional Review Board (IRB) (#Pro00030334). This clinical trial was registered with ClinicalTrials.gov (#NCT03762772) https://clinicaltrials.gov/ct2/show/NCT03762772?term=NCT03762772&draw=2&rank=1.

### Sample size and statistical methods

For this first-in-human Phase I study, sample size was based primarily on feasibility, rather than statistical considerations. There was no imputation of missing data. In general, continuous variables were summarized to indicate the study population sample size (N), number of participants with available data (n), mean, standard deviation (SD), median, 25th percentile, 75th percentile, minimum, and maximum values. Summaries of PK concentrations also included the geometric mean, geometric coefficient of variation (CV [%]), and number and percentage of participants with below the level of quantification (BLQ) concentrations. PK values of BLQ were imputed as ½ the lower limit of quantification (LLOQ). Summaries of PD endpoints included the 95% confidence interval of the mean and change from baseline p-value. Categorical variables were summarized by the population size (N), number of participants with available data (n), number of participants in each category, and the percentage of participants in each category. Unless otherwise noted, the denominator to determine the percentage of participants in each category was based on the number of participants with available data. Select ordinal data may have been summarized using both descriptive statistics and counts and percentages of participants in each category, as appropriate.

Baseline was the last non-missing value obtained closest to and prior to the insertion of the TAF/EVG vaginal insert. Data collection for baseline variables included demographic information including sexual and contraceptive history, medical history, pelvic examination, vital signs (height, weight and blood pressure), hematology testing, non-fasting chemistry testing, urine pregnancy testing, genital samples including vaginal and ectocervical tissue samples, and acceptability questionnaire. Safety assessments were performed throughout the study. TEAE data was collected at every visit after baseline sampling at visit 2. Normality of continuous data were tested using the PROC univariate command in SAS, then examining the normal quantile plot distribution and the Shapiro Wilk test statistic.

Paired comparisons measuring the change in an endpoint from baseline to post treatment were performed using a paired t test or Wilcoxon signed rank sum test on difference variables. We correlated continuous PK endpoints with continuous PD endpoints using a Spearman or Pearson correlation coefficient depending on the distribution of the data. We used multiple linear regression model to fit a model of the PD data with multiple PK endpoints. For the vaginal fluid PK/PD correlations, the model was fit to include HIV inhibition in vaginal fluid with vaginal fluid concentrations of EVG, TFV and TAF. In this model, the interaction between EVG and TFV was also included. For cervical vaginal tissue PK/PD correlations, p24 antigen production in ectocervical tissue was regressed over EVG, TFV, TFVDP and TAF concentrations in vaginal tissue. Statistical significance was determined at the level of alpha = 0.05.

## Results

### Patient disposition

**Figure 1:**
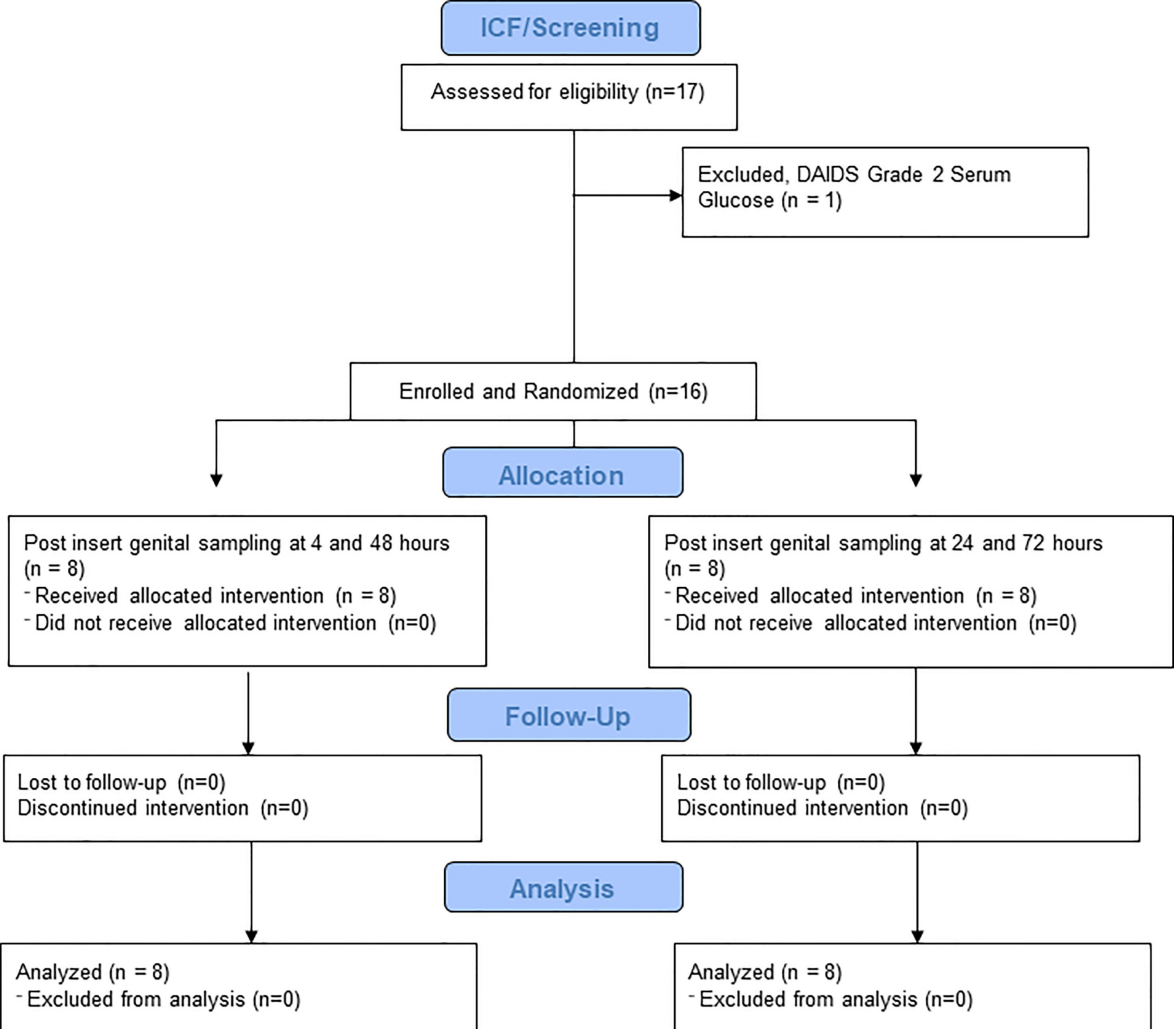
CONRAD A18-146 Flow Diagram. This figure demonstrates the number of volunteers screened, enrolled, randomized and completed.

We screened the first participant in December 2018 and the last participant visit took place in March 2019. As shown in **Figure 1**, 17 women provided informed consent and were screened and 16 women were enrolled and completed all study visits. **Table 1** demonstrates the demographics of the two sampling cohorts. Subjects reporting more than one race and more than one contraceptive method were counted separately in each category.

**Table 1:** CONRAD A18-146 demographics.

	Group 1 (Sampling 4 and 48 hours post dosing) (N=8)	Group 2 (Sampling 24 and 72 hours post dosing) (N=8)	Total (N=16)
Age in years Mean ±SD	39.0 ±4.72	40.5 ±7.39	39.8 ±6.04
Ethnicity (N, % of Group)			
Hispanic or Latino	1 (12.5%)	2 (25.0%)	3 (18.8%)
Not Hispanic or Latino	7 (87.5%)	6 (75.0%)	13 (81.3%)
Race (N, % of Group)			
American Indian or Alaska Native	1 (12.5%)	0 (0.0%)	1 (6.3%)
Asian	1 (12.5%)	0 (0.0%)	1 (6.3%)
Black or African American	2 (25.0%)	4 (50.0%)	6 (37.5%)
White	5 (62.5%)	4 (50.0%)	9 (56.3%)
Contraceptive Method Used On-Study (N, % of Group)			
Hormonal Methods, Except Vaginal Rings and DMPA	2 (25.0%)	2 (25.0%)	4 (25.0%)
Sterilization of Participant	2 (25.0%)	3 (37.5%)	5 (31.3%)
Abstinence	3 (37.5%)	1 (12.5%)	4 (25.0%)
Consistent Use of Condoms	1 (12.5%)	2 (25.0%)	3 (18.8%)
Sexual Partner Status (N, % of Group)			
Living with Partner	5 (62.5%)	4 (50.0%)	9 (56.3%)
Not Living with Partner	1 (12.5%)	3 (37.5%)	4 (25.0%)
No Partner	2 (25.0%)	1 (12.5%)	3 (18.8%)
Participant Ever Pregnant (N, % of Group)			
Yes	8 (100.0%)	7 (87.5%)	15 (93.8%)
No	0 (0.0%)	1 (12.5%)	1 (6.3%)
Time Since Pregnancy (years)			
Mean ±SD	9.0 ± 4.86	12.6 ± 7.11	10.6 ± 6.08

### Safety

Overall, there were 8 TEAEs reported by 7 participants (43.8%) (**Table 2**). The most common TEAE was increased non-fasting blood glucose, occurring in 3 (18.8%) participants overall. All non-fasting blood glucose measurements were categorized according to the NIH DAIDS grading table as Grade 1, or < 160 mg/dL. No other TEAE occurred in more than 1 participant. All TEAEs were mild (Grade 1), and none were assessed to be related to study treatment by the study investigators or medical monitor (**Table 2**). TEAEs considered related to study procedure (Grade 1 pain or discomfort related to genital biopsy procedure) occurred in 2 (12.5%) participants overall. The incidence of TEAEs was higher in Group 1 compared to Group 2; all events of increased blood glucose and events related to the study procedure occurred in Group 1 (3 [37.5%] and 2 [25.0%], respectively). No TESAEs, deaths or TEAEs led to dose interruption, discontinuation of study treatment, or premature withdrawal from the study.

**Table 2:** Treatment emergent adverse events in the CONRAD A18-146 study.

	Group 1* (N=8)	Group 2* (N=8)	Total (N=16)
Total Number of Treatment Emergent Adverse Events	7	7	8
Number (%) of Participants Reporting at Least One:			
TEAE	6 (75.0%)	1 (12.5%)	7 (43.8%)
TEAE by DAIDS Grade^[1]^			
Grade 1: Mild	6 (75.0%)	1 (12.5%)	7 (43.8%)
Grade 2 - 5: Moderate to Death	0 (0.0%)	0 (0.0%)	0 (0.0%)
TEAE by Relationship to Study Treatment^[2]^			
Not Related	6 (75.0%)	1 (12.5%)	7 (43.8%)
Related	0 (0.0%)	0 (0.0%)	0 (0.0%)
TEAE by Relationship to Study Procedure^[2]^			
Not Related	4 (50.0%)	1 (12.5%)	5 (31.3%)
Related	2 (25.0%)	0 (0.0%)	2 (12.5%)
TEAE Leading to Discontinuation of Study TX	0 (0.0%)	0 (0.0%)	0 (0.0%)
TEAE Requiring Dose Interruption of Study TX	0 (0.0%)	0 (0.0%)	0 (0.0%)
TEAE Leading to Premature Withdrawal from the Study or Death	0 (0.0%)	0 (0.0%)	0 (0.0%)

*Participants randomized to sampling at 4 and 48h (Group 1) or 24 and 72h (Group 2) post dosing.

AIDS, acquired immunodeficiency syndrome; DAIDS, Division of AIDS; TEAE, treatment-emergent adverse event.

^[1]^ Participants reporting more than one adverse event were counted only once using the highest DAIDS grade.

^[2]^ Participants reporting more than one adverse event were counted only once using the closest relationship to study treatment/procedure.

### Concentrations of EVG, TAF and TFV in plasma

Systemic concentrations of EVG were low but detectable in plasma at 4 hours post-dose, were higher at 24 hours post-dose (mean 0.33 ng/mL), and then were markedly lower by 48 hours and 72 hours post-dose. At 72 hours post-dose, 6 of 7 participants had BLQ plasma EVG levels. Similarly, the mean plasma concentration of the prodrug TAF was highest at 4 hours post-dose (mean 1.03 ng/mL), then lower by 24 hours (4 of 8 participants BLQ), 48 hours (7 of 8 participants BLQ), and 72 hours (7 of 7 participants BLQ) post-dose. Levels of TFV were detectable in plasma for most participants by 4 hours post-dose. TFV concentrations were higher at 24 hours post-dose (mean 0.48 ng/mL), decreasing by 48 hours and 72 hours post dose.

### Concentrations of EVG, TAF and TFV in vaginal fluid

**Figure 2:**
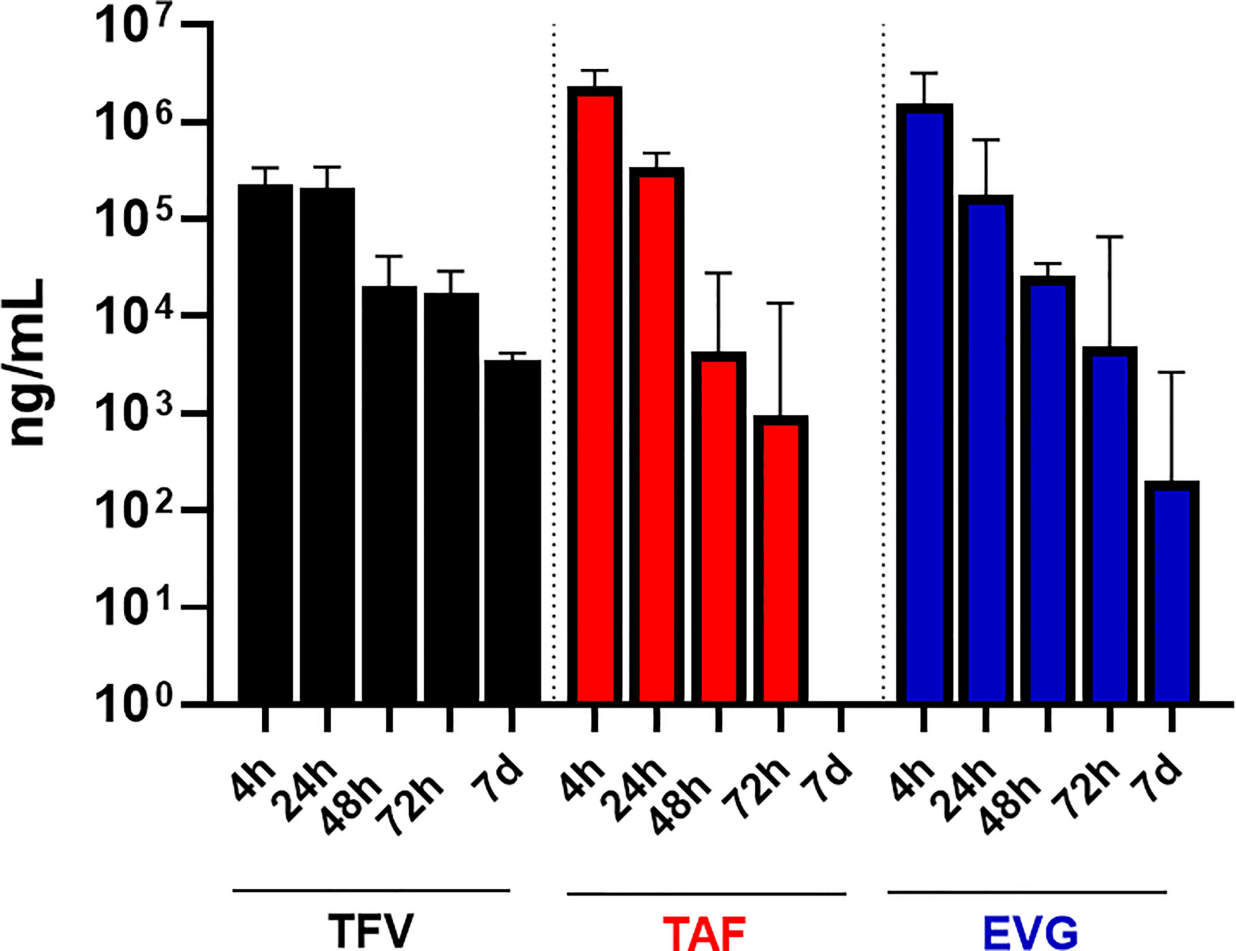
TFV, TAF and EVG in Vaginal Fluid. Black = TFV concentrations in ng/mL; Red = TAF concentrations in ng/mL; Blue = EVG concentrations in ng/mL.

One participant in Group 2 was not included because her pre and post vaginal fluid sample weights were not provided to the PK central laboratory due to a temporary scale malfunction, thus preventing calculation of fluid volume. As shown in **Table 3** and **Figure 2**, the median EVG vaginal fluid concentration was highest 4 hours post-dose (median 1,507,362 ng/mL), declining later and being approximately 7-fold lower by 24 hours post-dose and 12-fold lower by 48 hours post-dose. The concentration at 72 hours post-dose was in the same range as that of the 48 hour post dose. No participants had BLQ EVG levels in vaginal fluid from 4 hours through 72 hours post-dose. At 7 days post-dose, all but one participant had detectable EVG levels in vaginal fluid, and the median vaginal fluid levels of EVG were markedly lower relative to the 48 and 72 hour post-dose values.

**Table 3:** Concentrations of EVG, TAF and TFV (ng/mL) in vaginal fluid.

Analyte	Visit	Statistical Measures *	Group 1 4 hours at Visit 3 and 48 hours at Visit 4 (N=8)	Group 2 24 hours at Visit 3 and 72 hours at Visit 4 (N=8)
EVG (ng/mL) vaginal fluid	Visit 3	n	8	7
		Mean (SD)	2,201,502 (1,741,831)	323,378 (278,363)
		Median (Q1, Q3)	1,507,362 (1,054,818, 2,802,391)	177,765 (95,935, 649,951)
		Number (%) with BLQ	0 (0.0%)	0 (0.0%)
	Visit 4	n	8	7
		Mean (SD)	25,668 (14,044)	31,204 (52,647)
		Median (Q1, Q3)	26,090 (16,037, 33,252)	4,816 (1,535, 65,403)
		Number (%) with BLQ	0 (0.0%)	0 (0.0%)
	Visit 5	n	8	8
	[7 days post-dose]	Mean (SD)	923 (1,915)	1,804 (2,453)
		Median (Q1, Q3)	199 (37, 657)	376 (38, 3,556)
		Number (%) with BLQ	0 (0.0%)	1 (12.5%)
TAF (ng/mL) vaginal fluid	Visit 3	n	8	7
		Mean (SD)	3,662,350 (4,966,790)	368,539 (251,283)
		Median (Q1, Q3)	2,319,007 (1,267,333, 3,249,263)	340,854 (232,508, 474,736)
		Number (%) with BLQ	0 (0.0%)	0 (0.0%)
	Visit 4	n	8	7
		Mean (SD)	12,471 (15,094)	5,380 (6,788)
		Median (Q1, Q3)	4,278 (122, 27,786)	953 (16, 13,608)
		Number (%) with BLQ	0 (0.0%)	1 (14.3%)
	Visit 5	n	8	8
	[7 days post-dose]	Mean (SD)	10 (17)	22 (51)
		Median (Q1, Q3)	0.76 (0.01, 19.06)	0.79 (0.01, 13.50)
		Number (%) with BLQ	4 (50.0%)	3 (37.5%)
TFV (ng/mL) vaginal fluid	Visit 3	n	8	7
		Mean (SD)	241,795 (155,893)	227,198 (174,859)
		Median (Q1, Q3)	227,644 (142,779, 332,234)	210,543 (60,496, 342,268)
		Number (%) with BLQ	0 (0.0%)	0 (0.0%)
	Visit 4	n	8	7
		Mean (SD)	28,511 (19,961)	21,674 (18,390)
		Median (Q1, Q3)	19,961 (14,608, 37,790)	17,098 (9,004, 28,946)
		Number (%) with BLQ	0 (0.0%)	0 (0.0%)
	Visit 5:	n	8	8
	[7 days post-dose]	Mean (SD)	4,353 (3,645)	2,919 (2,386)
		Median (Q1, Q3)	3,592 (2,484, 4,668)	2,651 (1,052, 4,059)
		Number (%) with BLQ	0 (0.0%)	0 (0.0%)

*Means and Medians do not include BLQ values.

Similar to EVG, the median vaginal fluid concentration of the prodrug TAF was highest at 4 hours post-dose (median 2,319,007 ng/mL), decreased by approximately 10-fold at 24 hours post-dose, and was markedly lower by 48 and 72 hours post-dose. By 7 days post-dose, 7 of 16 participants had BLQ TAF levels in vaginal fluid and median TAF levels were markedly lower relative to the 48- and 72-hour post dose measurements (**Table 3**; **Figure 2**).

Finally, median vaginal fluid concentration of TFV was highest at 4 hours post-dose (median 227,644 ng/mL), maintaining similar concentrations at 24 hours post-dose. By 48 hours post-dose, the median TFV levels in vaginal fluid were approximately 8-fold lower than the 24-hour post dosing concentrations, showing an additional slight (less than 2-fold) decrease by 72 hours post-dose. At 7 days post-dose, TFV levels in vaginal fluid remained detectable for all participants (**Table 3**; **Figure 2**).

### Concentrations of TFV, TAF, EVG and TFV-DP in vaginal tissue

**Figure 3:**
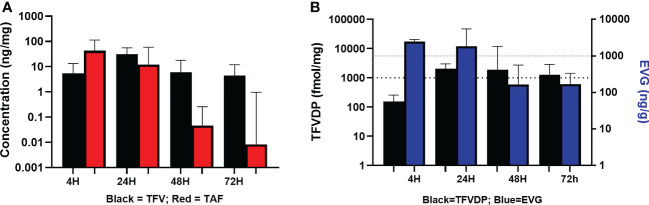
Concentrations of Analytes in Vaginal Tissue. **(A)** TFV and TAF Concentrations in Vaginal Tissue. Black = TFV in ng/mL; Red = TAF in ng/mL. **(B)** EVG and TFV-DP Concentrations in Vaginal Tissue. Black = TFV-DP in fmol/mg; Blue = EVG in ng/g.

As shown in **Table 4** and **Figures 3A, B**, median vaginal tissue concentration of the prodrug TAF was highest at 4 hours post-dose (median 42.87 ng/mg). TAF levels in vaginal tissue decreased by approximately 3-fold at 24 hours post dosing, being markedly lower by 48 hours (4 of 8 participants BLQ) and 72 hours (4 of 7 participants BLQ) post dose (**Table 4**; **Figure 3A**).

**Table 4:** Concentrations of EVG, TAF and TFV (ng/mg) and TFV-DP (fmol/mg) in vaginal tissue.

Analyte	Visit	Statistical Measures*	Group 1 4 hours at Visit 3 and 48 Hours at Visit 4 (N=8)	Group 2 24 hours at Visit 3 and 72 Hours at Visit 4 (N=8)
EVG (ng/mg)	Visit 3	n	8	7
		Mean (SD)	20.64 (15.11)	25.43 (23.57)
		Median (Q1, Q3)	17.75 (14.90, 20.03)	15.79 (2.84, 49.13)
		Number (%) with BLQ	0 (0.0%)	0 (0.0%)
	Visit 4	n	8	7
		Mean (SD)	1.60 (2.10)	1.05 (1.19)
		Median (Q1, Q3)	0.60 (0.27, 2.43)	0.62 (0.30, 1.67)
		Number (%) with BLQ	0 (0.0%)	0 (0.0%)
TAF (ng/mg)	Visit 3	n	8	7
		Mean (SD)	69.15 (85.49)	22.78 (28.15)
		Median (Q1, Q3)	42.87 (1.05, 112.99)	11.92 (0.41, 59.25)
		Number (%) with BLQ	0 (0.0%)	0 (0.0%)
	Visit 4	n	8	7
		Mean (SD)	0.15 (0.200)	0.48 (0.611)
		Median (Q1, Q3)	0.05 (0.02, 0.23)	0.01 (0.00, 0.98)
		Number (%) with BLQ	4 (50.0%)	4 (57.1%)
TFV (ng/mg)	Visit 3	n	8	7
		Mean (SD)	8.77 (7.378)	29.69 (25.049)
		Median (Q1, Q3)	5.42 (3.73, 12.67)	31.38 (6.64, 56.15)
		Number (%) with BLQ	0 (0.0%)	0 (0.0%)
	Visit 4	n	8	7
		Mean (SD)	9.61 (8.092)	6.66 (5.222)
		Median (Q1, Q3)	5.99 (3.27, 17.63)	4.51 (2.38, 11.95)
		Number (%) with BLQ	0 (0.0%)	0 (0.0%)
TFV-DP (fmol/mg)	Visit 3	n	8	7
		Mean (SD)	222.13 (256.72)	3642.08 (5557.17)
		Median (Q1, Q3)	156.80 (50.66, 257.97)	2040.79 (489.56, 3022.31)
		Number (%) with BLQ	0 (0.0%)	0 (0.0%)
	Visit 4	n	8	7
		Mean (SD)	5325.38 (5795.41)	2161.36 (2410.96)
		Median (Q1, Q3)	1934.10 (741.09, 11627.59)	1291.08 (888.06, 2875.39)
		Number (%) with BLQ	0 (0.0%)	0 (0.0%)

*Means and Medians do not include BLQ values.

TFV was detected in vaginal tissue by 4 hours post-dose, and was higher at 24 hours post-dose (median 31.38 ng/mg). By 48 and 72 hours post dose, concentrations were similar to the 4 hour levels. Vaginal tissue TFV levels were detectable in all participants at all time points (**Table 4**; **Figure 3A**).

Median vaginal tissue levels of EVG were slightly higher at 4 hours post-dose (median 17.75 ng/mg) relative to levels at 24 hours post-dose (median 15.79 ng/mg), and then markedly lower at 48 hours and 72 hours post-dose. EVG was detectable in all participants at all time points. All participants had vaginal tissue EVG concentrations above our *in vitro* estimated tissue benchmark for protection of 1000 ng/g (1 ng/mg) at 4 hours and 24 hours post dosing (**Table 4**; **Figure 3B**). Three of 8 participants at 48 hours post-dose and 2 of 7 participants at 72 hours post-dose continued to have vaginal tissue EVG concentrations above this PK benchmark (**Table 4**; **Figure 3B**).

Concentrations of TFV-DP were detected in vaginal tissue by 4 hours post-dose (median 156.80 fmol/mg) and were approximately 16-fold higher by 24 hours post-dose (median 2040.79 fmol/mg). TFV-DP concentrations decreased slightly (less than 2-fold) by 48 hours post dose and then were similar to the 24 hour level at 72 hours post dose. Vaginal tissue TFV-DP levels were detectable in all participants at all time points. Except at 4 hours post-dose (range 37.5-808.6 fmol/mg), median TFV-DP concentrations exceeded the target benchmark of 1000 fmol/mg of tissue at each of the subsequent time points including 72 hours post-dose. (**Table 4**; **Figure 3B**).

### Inhibition of HIV-1 and HSV-2 *in vitro* by vaginal fluids

**Figure 4:**
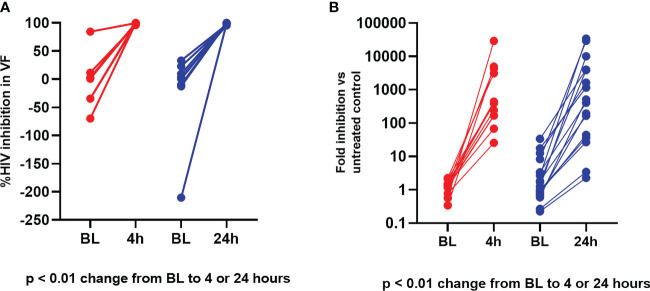
Ex vivo PD modeling of anti-viral effect in vaginal fluid. **(A)** Vaginal Fluid HIV-1 Inhibition. Red = Baseline (BL) to 4 hours; Blue = Baseline to 24 hours. **(B)** Vaginal Fluid HSV-2 Inhibition. Red = Baseline (BL) to 4 hours; Blue = Baseline to 24 hours.

For anti-HIV percent inhibition in vaginal fluid, median values at baseline were similar between the time point groups (p = 0.75). In addition, at post treatment visits, median HIV-1 and HSV-2 inhibition by vaginal fluid was similarly high whether obtained at 4 hours post dosing (Group 1) versus 24 hours post dosing (Group 2) (p = 0.83). As shown in **Figures 4A, B**, there was a statistically significant increase from baseline in vaginal fluid inhibition of HIV-1 and HSV-2 post treatment for both sampling time points (p values < 0.01).

### Viral p24 antigen production after ex vivo infection of ectocervical tissue biopsies with HIV-1

As shown in **Table 5**, we found a statistically significant decrease from baseline in p24 antigen production (all p values < 0.01) 4 hours after insert administration, demonstrating specific antiviral activity and protection from infection.

**Table 5:** Ectocervical p24 antigen production after ex vivo HIV infection at baseline and 4 hours post dosing.

P24 antigen production variable (pg/mL)	Baseline Visit 2 (n = 16 ectocervical biopsies among 8 participants)			Post Treatment Visit 3 (4 hours post dosing for Group 1) (n = 16 ectocervical biopsies among 8 participants)			P value
	Mean	SD	Median	Mean	SD	Median	
p24 Area Under Curve	4697.19	10830.75	380.9	106.5	18	102	< 0.01
p24 Cumulative Production	1687.58	3577.12	205.72	39	12	36	< 0.01
p24 Day 21 of tissue culture	594.7	934.84	124.14	9	12	6	< 0.01

### Viral replication after ex vivo infection of ectocervical tissue biopsies with HSV-2

The quantity of HSV-2 DNA detected at days 3, 6, 9 and 12 of cell culture and cumulative HSV-2 DNA detected are shown in **Supplemental Table 2**. Median values at end of culture (day 12) and cumulative were lower after treatment (24 hours post dose) but changes from baseline post-dosing were not statistically significant (all p values > 0.80).

### Correlation between drug concentrations (PK) and *in vitro* HIV inhibition (PD) in vaginal fluid

There were significant simple linear correlations (all p values < 0.01) between all vaginal fluid PK concentrations and HIV inhibition by vaginal fluid (Spearman correlation coefficients for TFV, TAF and EVG versus percent HIV inhibition were 0.76, 0.78 and 0.81, respectively). Using multiple linear regression model (TAF + TFV or EVG), TFV in vaginal fluid remained significant (p < 0.01) and positively predictive of HIV inhibition. There was a significant interaction between vaginal fluid TFV and vaginal fluid EVG (p = 0.04) in multiple regression analysis, indicating both drugs contribute to the observed effect.

In vaginal tissue, all PK concentration endpoints (TFV, TFV-DP, EVG and TAF) were significantly and negatively correlated with ectocervical HIV p24 antigen production (p24 area under the curve, p24 cumulative production and p24 at day 21 of tissue culture) with Spearman correlation coefficients ranging from -0.42 to -0.60 and all p values < 0.01.

### Evaluation of disintegration time and acceptability of the vaginal insert

The vaginal insert was completely dissolved and absorbed into the mucosal tissues with no remnants visible for 12 (75%) participants at the earliest assessment (4 or 24 hours). For the remaining 4 participants, the insert was dissolved but there were small clear gel-like areas of spreading observed. No participant had an intact vaginal insert at the first dissolution assessment.

Most participants (13, [81%]) had not previously participated in a vaginal insert study. Overall, at baseline, most participants stated they were very comfortable with inserting products into their vagina (13 [81.3%]), despite the fact that most had not previously used vaginal suppositories or inserts (12 [75.0%]). When asked their first impressions of the insert based on seeing it, all participants expressed either no preference or liked how the insert looked (16 [100%]). Open-ended responses describing first impressions included statements that it looked like a pill or bullet, and others stated it was small and/or smaller than expected. The term insert was associated with a bigger item, similar to a tampon. Two participants thought the insert looked hard and uncomfortable. Most participants at baseline stated they would be somewhat or very interested in using the product in the future if it protected against HIV-1 and HSV-2 (14 [87.5%]), which stayed consistent after using the vaginal insert in the trial. At the post treatment visit, most participants found the insert very or somewhat acceptable in size (16 [100%]), ease of insertion (16 [100%]), comfort after insertion (15 [93.8%]), dissolvability (15 [93.8%]), residue (13 [81.3%]), leakage (14 [87.5%]), scent (15 [93.8%]), color (16 [100%]), and discreetness (15 [93.8%]) (**Table 6**). While acceptability of the all insert traits was high, dissolvability, residue and leakage were a little less acceptable for some participants. Seven participants overall noticed abnormal leakage/discharge either less than an hour (3 [18.8%]) or 1 to 4 hours after dosing (4 [25.0%]) by feeling it in their underwear (6 [37.5%]). Most of the open ended responses regarding leakage indicated it was a small amount of whitish discharge with no odor. Just under half of participants overall (7 [43.8%]) reported that using an applicator would make insertion easier. Overall, participants generally were either unsure of the time it took for the insert to dissolve (7 [43.8%]) or reported less than 2 hours (7 [43.8%]). When asked the ideal dissolution time, 9 [56.3%] participants preferred less than 30 minutes and 5 [31.3%] reported no preference. All participants (16 [100%]) reported it was possible to use the insert without their partner’s knowledge. Most participants (15 [93.8%]) reported not feeling anything once the insert was in the vagina. Additional responses regarding product characteristics are noted in **Table 6**.

**Table 6:** Acceptability of insert characteristics.

Vaginal Insert Characteristics (N=16)	ACCEPTABLE			UNACCEPTABLE		
	Very	Somewhat	A little	A little	Somewhat	Very
Size	16 (100.0%)	0 (0.0%)	0 (0.0%)	0 (0.0%)	0 (0.0%)	0 (0.0%)
Ease of inserting into vagina	15 (93.8%)	1 (12.5%)	0 (0.0%)	0 (0.0%)	0 (0.0%)	0 (0.0%)
Comfort after it was inserted	15 (93.8%)	0 (0.0%)	1(12.5%)	0 (0.0%)	0 (0.0%)	0 (0.0%)
Dissolvability (rate and amount)	12 (75.0%)	3 (18.8%)	1 (12.5%)	0 (0.0%)	0 (0.0%)	0 (0.0%)
Residue (amount, color, texture)	11 (68.8%)	2 (25.0%)	1 (12.5%)	1 (12.5%)	1 (12.5%)	0 (0.0%)
Leakage	11 (68.8%)	3 (18.8%)	1 (12.5%)	1 (12.5%)	0 (0.0%)	0 (0.0%)
Scent	15 (93.8%)	0 (0.0%)	1 (12.5%)	0 (0.0%)	0 (0.0%)	0 (0.0%)
Color	16 (100.0%)	0 (0.0%)	0 (0.0%)	0 (0.0%)	0 (0.0%)	0 (0.0%)
Discreetness	14 (87.5%)	1 (12.5%)	0 (0.0%)	1 (12.5%)	0 (0.0%)	0 (0.0%)

## Discussion

We found that a single vaginal application of a TAF/EVG containing insert was safe, well tolerated and acceptable. There were no product related TEAEs. The insert rapidly delivered TAF, which converted into TFV, and EVG, yielding high concentrations in vaginal fluid by 4h after dosing (first time point evaluated) and leading to high tissue concentrations of EVG at 4 and 24h post dosing, followed by high TFV-DP, TFV active metabolite, concentrations 24-72h post dosing. Altogether these PK data were in agreement with the significant PD antiviral effect observed both in vaginal fluid and tissue.

Data from several recent acceptability studies and discrete choice experiments using placebo products (Nel et al., 2011; Luecke et al., 2016; Montgomery et al., 2019) support that a discreet, on demand, topical method would fill an important niche in the current HIV-1 prevention armamentarium for women. Recent acceptability data from the contraceptive field, among women in both high and low income countries, indicate that many AGYW prefer an on demand, female controlled, peri-coital contraceptive (Halpern et al., 2014; Raymond et al., 2011; Halpern et al., 2008; Sarkar, 2011; Cover et al., 2013b; Cahill and Blumenthal, 2018), rather than a daily regimen or even a long acting regimen. Importantly, one study demonstrated that the pregnancy prevention efficacy of on-demand oral levonorgestrel was similar to that seen with typical use of daily oral contraceptive pills (Halpern et al., 2014). Key stakeholders and health care providers also agree that an event-driven method fills an important gap in women’s reproductive health products (Cover et al., 2013a). An on-demand topical prevention product has the potential to provide greater flexibility to meet end user’s needs, potentially leading to increased overall uptake. Owing to their low plasma/systemic drug levels, topical on-demand products exhibit less side effects, increasing tolerability and acceptability.

The TAF/EVG insert is more discreet and convenient than the TFV vaginal gel, an extensively tested (Karim et al., 2010; Delany-Moretlwe et al., 2018) topical, peri-coital microbicide. The 4 mL of vaginal gel, which required two doses, resulted in vaginal discharge and wetness that was desirable to some women but unacceptable to many others (Mayer et al., 2006; Rosen et al., 2008; Mehendale et al., 2012; Minnis et al., 2013). As compared to the pre-filled TFV vaginal gel doses, multiple doses of the vaginal insert can be packaged in a small discreet container, which would likely be more desirable to AGYW who may live in cramped quarters with minimal privacy. An on-demand, prevention product may also be appealing to end users who have infrequent intercourse, or who do not want to be on a daily or long-acting prevention regimen (Halpern et al., 2014; Taylor et al., 2014). An on-demand prevention product that women could use pre- or post-coitally, with an extended window of protection, would also be a safe alternative for women who are in less stable relationships, where use of a daily regimen, short acting regimen may decrease their ability to use a product covertly, which may increase their risk of intimate partner violence (Giovenco et al., 2022; Jeffers et al., 2022).

As expected, the topical product resulted in low systemic absorption. Systemic TFV concentrations were consistent with past studies of peri-coital topical TFV vaginal gel (Schwartz et al., 2011; Hendrix et al., 2013; Herold et al., 2016; Robinson et al., 2018) and TFV vaginal film (Bunge et al., 2018; Robinson et al., 2018) and were lower than observed with the continuous use TFV releasing intravaginal ring (IVR) (Thurman et al., 2018). Systemic TFV exposure after the topical insert was also significantly lower than with oral dosing (Dumond et al., 2007; Patterson et al., 2011; Hendrix et al., 2013; Ruane et al., 2013; Donnell et al., 2014; Hendrix et al., 2016), even after a single oral dose (Patterson et al., 2011). Although low systemic drug exposure has the advantages of reducing systemic side effects, there is a theoretical concern that it may increase the risk of acquiring a resistant HIV-1 mutation if an infected individual uses topical PrEP. This theoretical risk, however, has not been proven to be a reality in large clinical trials or preclinical studies in non-human primates using TFV-containing topical products (Karim et al., 2010; Dobard et al., 2012; Dobard et al., 2015; Marrazzo et al., 2015; Delany-Moretlwe et al., 2018), and currently, the benefits of vaginally or rectally applied PrEP outweigh this risk.

A single topical dose delivered high local mucosal concentrations of TAF, TFV and EVG for up to 72 hours post dosing. All but one participant had detectable vaginal concentrations of TFV and EVG for up to 7 days post dosing. TAF is a prodrug of TFV which rapidly partitions into tissues and in particular immune cells (Callebaut et al., 2015), and as expected, did not remain in the genital fluid for as long as TFV and EVG, with about half of participants having concentrations below the level of detection by 7 days post dosing. PK benchmark surrogates of protection against HIV-1 for TFV containing topical microbicides are still often modeled by concentrations of TFV in the CV aspirate and its association with HIV seroconversion in the TFV gel CAPRISA 004 study (Kashuba et al., 2015). A TFV concentration over 100 ng/mL in the CV aspirate conferred an estimated 65% protection against HIV-1 acquisition, while a CV aspirate TFV concentration of over 1,000 ng/mL provided an estimated 76% protection against HIV-1 (Karim et al., 2011; Kashuba et al., 2015). We found that mean and median concentrations of vaginal fluid TFV exceeded 1,000 ng/mL as far out as 7 days post dosing, peaking at the first sampling, 4 hours post dosing, at a median of over 200,000 ng/mL. EVG also showed high concentrations in CVF, several times higher than its IC90 (45ng/mL) (Ramanathan et al., 2011; Elliot et al., 2016) at 4h post dose.

As expected, we found significant positive linear correlations between vaginal fluid inhibition of HIV *in vitro* and vaginal fluid concentrations of TAF, EVG and TFV. The inhibitory activity of the vaginal fluid against HIV-1 at baseline was similar to previous data in healthy women (Keller et al., 2011; Chappell et al., 2015; Thurman et al., 2016; Thurman et al., 2018). Consistent with the high vaginal concentrations of TFV, once participants used the vaginal insert, the inhibitory activity of vaginal fluid against both HIV-1 and HSV-2 *in vitro* increased significantly, similarly to levels seen with use of TFV vaginal gel (Keller et al., 2011; Herold et al., 2014; Schwartz et al., 2014; Thurman et al., 2018). It is noteworthy that the inhibitory activity of vaginal fluid against both HIV-1 and HSV-2 was high and similar at 4 and 24 hours post dosing, supporting an extended window of protection.

Similar to the vaginal fluid PK data, we found peak concentrations of the TFV prodrug, TAF, in tissue, at 4 hours post dosing. Concentrations of TFV, EVG and the active metabolite, TFV-DP, in vaginal tissue peaked at 24 hours post dosing. This study is limited by the fact that post dosing tissue PK samples were collected at 4 hours, 24 hours, 48 hours and 72 hours post dosing, so we cannot determine if these tissue concentrations peaked before or between 4 and 24 hours post dosing. However, data in non-human primates administered with the same insert vaginally show high levels of EVG and TAF/TFV in CV fluid and tissues two hours after insertion, the earliest time point studied (Dobard et al., 2022). The fact that systemic plasma concentrations in women were detectable, albeit low, by 4 hours post use supports that local absorption was underway prior to the 4 hour time point. In a previous study of a TFV/emtricitabine vaginal insert we observed that drug release begins as soon as the insert starts disintegrating [ (Mauck et al., 2015) Because of its physicochemical properties, EVG partitions into the tissue quickly after insertion, which would confer rapid protection. Conversely, TAF requires conversion to TFV and then to TFV-DP intracellularly, which leads to a later peak (~24h after insertion) but prolonged high levels.

The benchmark of 1,000 fmol/mg for TFV-DP levels in tissue comes from a delayed efficacy study of TFV gel in macaques, demonstrating a drop in protection associated with TFV-DP mucosal levels below 1000 fmol/10^6^ cell (Dobard et al., 2012). Most participants in this study had TFV-DP tissue concentrations of 1,000 fmol/mg or higher after 4h post dosing.

Benchmark tissue concentrations have not been established for EVG in HIV-1 prevention, although its IC50 *in vitro* and PA IC95 are 0.4 ng/mL and 45 ng/mL, respectively (Sato et al., 2006; Ramanathan et al., 2011; Elliot et al., 2016). Our previous *ex vivo* modeling experiments, discussed above, support a target protective tissue EVG concentration of 1 ng/mg (Ouattara et al., 2016). It is worth noting that median tissue concentrations of EVG at 4 and 24 hours post dosing were >20 times higher than that threshold, and >400 times the PA IC95. Using cell based and CV tissue based assays, we demonstrated that a combination of TAF and EVG provided extended pre- and post-exposure inhibition of HIV replication. This concept has been tested and proven in a low-dose repeated SHIV challenge non-human primate (NHP) model where vaginal application of the TAF/EVG inserts was highly effective in preventing infection either following a pre-exposure or a post-exposure modality (Dobard et al., 2022). Furthermore, two inserts applied prior to rectal SHIV exposure were also highly effective in preventing infection (Makarova et al., 2022). Both NHP studies provide strong evidence of efficacy for the TAF/EVG inserts applied before or after viral exposure.

The likelihood of protection against mucosal HIV-1 acquisition is also supported by our clinical *ex vivo* modeling data. We found a significant reduction in HIV p24 antigen production from ectocervical tissues obtained 4 hours post dosing. Further, we found that all analytes (TFV, TFV-DP, EVG and TAF) were significantly and negatively correlated with p24 antigen production from ectocervical tissues treated *ex vivo* with HIV-1_BaL_. Furthermore, the different PK profiles of EVG and TFV-DP, the former peaking earlier and the latter lasting longer, are complementary and supportive of an extended window of protection. The PK profiles and *in vitro* and ex vivo PD modeling from this study, as well as pre-clinical animal data (Massud et al., 2015; Dobard et al., 2022) support that women would be protected after a single vaginal dose, for 2-3 days post dosing. Although a theoretical concern, existing data do not support the genesis of resistant virus after topical application of TFV or other anti-retrovirals (Wei et al., 2014; Marrazzo et al., 2015; Baxter et al., 2019; Steytler et al., 2022).

In addition, although not the primary outcomes of the trials, topical TFV gel reduced acquisition of genital HSV-2 compared to placebo in two previous Phase IIb trials (Abdool Karim et al., 2010; Marrazzo et al., 2014; Abdool Karim et al., 2015). In the CAPRISA 004 cohort, participants with CV aspirate concentrations of TFV of 10,000 ng/mL or higher had a 63% protection against HSV-2 compared to participants with no detectable TFV in CV aspirate (Abdool Karim et al., 2015). All participants in this study had vaginal fluid TFV concentrations exceeding 10,000 ng/mL between 4 and 48 hours post dosing. *In vitro* cell and tissue modeling studies support that the anti-HSV-2 activity of TFV becomes evident at concentrations of approximately 10,000 – 200,000 ng/mL (Andrei et al., 2011). We found median concentrations of TFV in vaginal fluid exceeded 200,000 ng/mL at both 4 and 24 hours post dosing and our *in vitro* PD modeling data was consistent, showing significant inhibition of HSV-2 by vaginal fluid at these time points. Despite high tissue TFV concentrations and reduction in HSV-2 replication after dosing, changes from baseline in HSV-2 DNA production in a recently developed exploratory *ex vivo* tissue infection assay were not statistically significant. We have since been refining this tissue infection protocol. It is possible that the tissue culture fluid may have diluted TFV concentrations locally below the concentration required to inhibit HSV-2 (Andrei et al., 2011).

The strengths of this study include longitudinal follow up with systemic and local PK characterization and PD modeling in this first-in-woman study of a vaginal insert containing two potent anti-retrovirals (ARVs). Other ARVs are being developed as vaginal inserts for HIV-1 prevention (reviewed in (Peet et al., 2019)). EVG has been tested as a nanoparticle intravaginal formulation in mice (Mohideen et al., 2017), but this is the first in woman study of a topical product containing TAF and EVG for HIV prevention.

Product adherence was confirmed in all participants, since the dose was self-administered in the clinic. Although the sample size was based on feasibility and not statistical calculations, participants demonstrated high tissue and fluid PK concentrations, which correlated well with PD modeling endpoints. Because the sample size was consistent with a Phase I, first-in-human clinical trial, we could only obtain tissue for PK and PD sampling at only a few times post dosing. However, these times provide important initial PK profiles for post dosing intervals. We only tested a single dose, and so we will need to perform follow on safety, PK and PD studies to understand the performance of the product after multiple exposures. Our current PK data likely support a 72-hour span of protection, but we will need to confirm this hypothesis with additional PD modeling at the 72-hour mark.

Acceptability data obtained for this low risk, Phase I study population may not be applicable to younger, at risk populations worldwide in different cultural contexts. While this study population was not the target end user, participants were interested in using the product outside of the trial if it protected from HIV and HSV, presumably due to the protection from HSV, which is the most common sexually transmitted infection world-wide. Phase I studies provide a good opportunity to detect major negative reactions to investigational products and discover certain product attributes that may need further refinement. We previously tested a vaginal insert containing tenofovir disoproxil fumarate and emtricitabine for HIV-1 prevention (CONRAD 117 (NCT01694407)) (Mauck et al., 2015), and found that although PK profiles were achieved, the disintegration of the insert was less than optimal and resulted in unacceptable vaginal discharge. We therefore re-formulated the inserts and assessed disintegration times and acceptability of 4 placebo vaginal inserts (in CONRAD 134, NCT02534779) (Littlefield et al., 2017) and selected the optimal insert to be used in the current study. While dissolvability, residue and leakage were a little less acceptable than other characteristics of the vaginal insert in this study, these characteristics would presumably change significantly with sexual intercourse and the increase of fluids. This study did not allow for sexual activity during dosing and testing. These are important factors to further explore in future studies, especially with the target end users. Overall, in this study, the inserts were found to be highly acceptable.

In conclusion, these data support that a single, easy to use vaginal insert delivers high mucosal concentrations of TAF, TFV, TFV-DP, and EVG locally for an extended time (3+ days) after dosing. Clinical PK and PD surrogates, in addition to NHP studies, support efficacy against HIV-1, and possibly HSV-2, at the CV mucosal level. Additionally the inserts were safe and acceptable, and dissolved rapidly. An on-demand, event-driven product, delivering highly potent ARVs, throughout an extended window of time, will fill an important gap in the current HIV prevention options, especially for AGYWs.

## Data availability statement

The original contributions presented in the study are included in the article/**Supplementary Material**. Further inquiries can be directed to the corresponding author.

## Ethics statement

The studies involving human participants were reviewed and approved by the Advarra Institutional Review Board (Pro00030334) and registered with ClinicalTrials.gov (#NCT03762772). The patients/participants provided their written informed consent to participate in this study.

## Author contributions

AT conducted the clinical trial, managed the participants, interpreted the data, and drafted and reviewed the manuscript. LO performed the anti-HIV activity experiments. NY performed the anti-HSV2 activity experiments. XF performed data analyses. PA and LB performed the pharmacokinetic analyses. HH analyzed the acceptability data. MC and OS supervised product development of the tablets. GD obtained funding, participated in product development and clinical study design. All authors contributed to the article and approved the submitted version.
