# Supplementary material for: A phase I study to assess safety, pharmacokinetics, and pharmacodynamics of a vaginal insert containing tenofovir alafenamide and elvitegravir
Source: Front Cell Infect Microbiol. 2023 Apr 19;13:1130101. doi: 10.3389/fcimb.2023.1130101 (PMC10154607; doi:10.3389/fcimb.2023.1130101)
Supplement: Supplementary file 1 [file DataSheet_1.docx]

**Supplemental Methods**

**Bioanalytical assay details for TAF, TFV, and EVG**

For TAF and TFV, an extraction procedure with acetonitrile is used to precipitate protein and an aliquot of the supernatant is dried under nitrogen and reconstituted in ammonium acetate buffer, pH 5 (5%MeCN) before analysis. The chromatographic separation is performed with a Luna Omega Polar C18 1.7µm particle size, 2.1x50mm column. A 1% to 50% acetonitrile gradient over 2 minutes is used to elute the compounds. The mobile phase A consists of H_2_O with 0.025% formic acid and mobile phase B is 50:50 H2O:acetonitrile with 0.05% formic acid. A Waters Acquity H-Class UPLC is coupled to a Waters Xevo TQS Micro triple quadrupole mass spectrometer. The detection and quantitation is achieved by protonated electrospray (ESI^+^) MS/MS detection. The resulting multiple reaction monitoring (MRM) for TFV/TFV-IS are 288.0→176.1 and 293.0→181.1, respectively. The resulting MRM for TAF/TAF-IS are 477.1→176.2 and 484.2→176.2, respectively. Two concentration ranges were utilized for standard curves for each compound with both utilizing weighted (1/X^2^) quadratic fit.

For EVG, plasma diluted with phosphate buffered saline (PBS) was subjected to a liquid-liquid extraction procedure with MTBE. The chromatographic separation is performed on a Waters XBridge BEH C18, 2.1x75 mm, reversed-phase column with a 2.5 micron particle size. The mobile phase consists of an isocratic flow of 75:25 acetonitrile:water with 0.1% formic acid (v:v) at 0.200 mL/min. An Accela HPLC is coupled to a Vantage triple quadrupole mass spectrometer (ThermoScientific). The detection and quantitation of EVG and EVG-IS is achieved by protonated electrospray (ESI^+^) MS/MS detection. The resulting SRM are 448.7→344.3 and 456.5→344.3, respectively.
