# Supplementary material for: A phase I study to assess safety, pharmacokinetics, and pharmacodynamics of a vaginal insert containing tenofovir alafenamide and elvitegravir
Source: Front Cell Infect Microbiol. 2023 Apr 19;13:1130101. doi: 10.3389/fcimb.2023.1130101 (PMC10154607; doi:10.3389/fcimb.2023.1130101)
Supplement: Supplementary file 2 [file Table_1.docx]

**Supplemental Table 1 |** Schedule of evaluations CONRAD A18-146 study

|  | | | **Screening/ Enrollment** | **Baseline** | **Single Dose TAF/EVG**  **Vaginal Insert** | | | | **Follow up** |
| --- | --- | --- | --- | --- | --- | --- | --- | --- | --- |
|  |  |  | **Visit 1** | **Visit 2** | **Visit 3**^1^ | | **Visit 4**^1^ | | **Visit 5** |
| **Sampling Time Point after Dosing** | | |  |  | **4 hours** | **24 hours** | **48 hours** | **72 hours** | **7d (±2 days)** |
| Informed consent medical history, vital signs | | | ✓ |  |  |  |  |  |  |
| **Urine** | Pregnancy Test | | ✓ | ✓ | ✓ | ✓ |  |  |  |
| **Blood** | HIV, HBsAg, HSV2 IgG | | ✓ |  |  |  |  |  |  |
|  | Safety (CBC, chemistries) | | ✓ |  |  |  | ✓ | ✓ |  |
|  | PK (Plasma): TAF, TFV, EVG | |  |  | ✓ | ✓ | ✓ | ✓ |  |
|  | Pelvic Exam | Gram Stain, STI testing | ✓ |  |  |  |  |  |  |
|  |  | Semen Test |  | ✓ | ✓ | ✓ | ✓ | ✓ | ✓ |
|  |  | Insert disintegration assessment |  |  | ✓ | ✓ |  |  |  |
|  | PK | CV Fluid  TAF, TFV, EVG |  |  | ✓ | ✓ | ✓ | ✓ | ✓ |
|  |  | CV Tissue - Biopsy  TAF, TFV, TFV-DP, EVG |  |  | ✓ | ✓ | ✓ | ✓ |  |
|  | PD | CV Fluid  anti-HIV and HSV2 activity |  | ✓ | ✓ | ✓ |  |  |  |
|  |  | CV Tissue – Biopsy^2^  HSV infectivity |  | ✓ |  | ✓ |  |  |  |
|  |  | CV Tissue-Biopsy^2^  HIV Infectivity |  | ✓ | ✓ |  |  |  |  |
| Randomization to sampling schedule^1^ | | |  | ✓ |  |  |  |  |  |
| Study product insertion in clinic | | |  |  | ✓ | ✓ |  |  |  |
| Acceptability questionnaire | | |  | ✓ | ✓ | ✓ |  |  |  |

(✓) = if indicated

^1^ **Time point Group 1**: post-dose sampling at 4 and 48 hours; **Time point Group 2**: post-dose sampling at 24 and 72 hours

^2^ Participants in Time point Group 1 had HIV Infectivity sampling collected at Visit 2 and Visit 3; Participants in Time point Group 2 had HSV infectivity sampling collected at Visit 2 and Visit 3
