# Supplementary material for: A phase I study to assess safety, pharmacokinetics, and pharmacodynamics of a vaginal insert containing tenofovir alafenamide and elvitegravir
Source: Front Cell Infect Microbiol. 2023 Apr 19;13:1130101. doi: 10.3389/fcimb.2023.1130101 (PMC10154607; doi:10.3389/fcimb.2023.1130101)
Supplement: Supplementary file 3 [file Table_2.docx]

**Supplemental Table 2 |** Change in HSV-2 DNA production from ectocervical tissue from baseline to 24 hours post treatment

| HSV2 DNA Production at day of tissue culture | Visit 2 Baseline (n = 16 ectocervical biopsies from 8 participants) HSV-2 DNA Production | | | | Visit 3, 24 hours post insert dosing (n = 16 ectocervical biopsies from 8 participants) HSV-2 DNA production | | | | P value (Wilcoxon Signed Rank Sum) |
| --- | --- | --- | --- | --- | --- | --- | --- | --- | --- |
|  | N | Mean | SD | Median | N | Mean | SD | Median |  |
| **HSV2 Day 3** | 16 | 18.38 | 30.72 | 6.56 | 16 | 27.37 | 45.28 | 11.44 | 0.80 |
| **HSV2 Day 6** | 16 | 82.73 | 198.55 | 16.74 | 16 | 81.91 | 192.82 | 15.89 | 1.00 |
| **HSV2 Day 9** | 16 | 506.8 | 1734.59 | 10.56 | 16 | 108.74 | 185.45 | 10.16 | 1.00 |
| **HSV2 Day 12** | 16 | 1612.72 | 5676.67 | 44.31 | 16 | 346.23 | 598.79 | 18.21 | 0.80 |
| **HSV2 Cumulative Production Days 3 - 12** | 16 | 2220.62 | 7607.34 | 127.25 | 16 | 564.25 | 865.59 | 102.02 | 1.00 |
